# Encapsulation of β-NGF in injectable microrods for localized delivery accelerates endochondral fracture repair

**DOI:** 10.3389/fbioe.2023.1190371

**Published:** 2023-05-22

**Authors:** Kevin O. Rivera, Darnell L. Cuylear, Victoria R. Duke, Kelsey M. O’Hara, Justin X. Zhong, Nafisa A. Elghazali, Joel A. Finbloom, Bhushan N. Kharbikar, Alex N. Kryger, Theodore Miclau, Ralph S. Marcucio, Chelsea S. Bahney, Tejal A. Desai

**Affiliations:** ^1^ Graduate Program in Oral and Craniofacial Sciences, School of Dentistry, University of California, San Francisco (UCSF), San Francisco, CA, United States; ^2^ Department of Orthopaedic Surgery, Orthopaedic Trauma Institute, University of California, San Francisco (UCSF), San Francisco, CA, United States; ^3^ Department of Bioengineering and Therapeutic Sciences, University of California, San Francisco (UCSF), San Francisco, CA, United States; ^4^ Center for Regenerative and Personalized Medicine, The Steadman Philippon Research Institute (SPRI), Vail, CO, United States; ^5^ UC Berkeley—UCSF Graduate Program in Bioengineering, San Francisco, CA, United States; ^6^ School of Dentistry, University of California, San Francisco (UCSF), San Francisco, CA, United States; ^7^ Department of Bioengineering, University of California, Berkeley (UC Berkeley), Berkeley, CA, United States; ^8^ School of Engineering, Brown University, Providence, RI, United States

**Keywords:** fracture repair, endochondral ossification, beta-nerve growth factor, drug delivery, sustained release, poly (ethylene) glycol dimethacrylate

## Abstract

**Introduction:** Currently, there are no non-surgical FDA-approved biological approaches to accelerate fracture repair. Injectable therapies designed to stimulate bone healing represent an exciting alternative to surgically implanted biologics, however, the translation of effective osteoinductive therapies remains challenging due to the need for safe and effective drug delivery. Hydrogel-based microparticle platforms may be a clinically relevant solution to create controlled and localized drug delivery to treat bone fractures. Here, we describe poly (ethylene glycol) dimethacrylate (PEGDMA)-based microparticles, in the shape of microrods, loaded with beta nerve growth factor (β-NGF) for the purpose of promoting fracture repair.

**Methods:** Herein, PEGDMA microrods were fabricated through photolithography. PEGDMA microrods were loaded with β-NGF and *in vitro* release was examined. Subsequently, bioactivity assays were evaluated in vitro using the TF-1 tyrosine receptor kinase A (Trk-A) expressing cell line. Finally, *in vivo* studies using our well-established murine tibia fracture model were performed and a single injection of the β-NGF loaded PEGDMA microrods, non-loaded PEGDMA microrods, or soluble β-NGF was administered to assess the extent of fracture healing using Micro-computed tomography (µCT) and histomorphometry.

**Results:** *In vitro* release studies showed there is significant retention of protein within the polymer matrix over 168 hours through physiochemical interactions. Bioactivity of protein post-loading was confirmed with the TF-1 cell line. *In vivo* studies using our murine tibia fracture model show that PEGDMA microrods injected at the site of fracture remained adjacent to the callus for over 7 days. Importantly, a single injection of β-NGF loaded PEGDMA microrods resulted in improved fracture healing as indicated by a significant increase in the percent bone in the fracture callus, trabecular connective density, and bone mineral density relative to soluble β-NGF control indicating improved drug retention within the tissue. The concomitant decrease in cartilage fraction supports our prior work showing that β-NGF promotes endochondral conversion of cartilage to bone to accelerate healing.

**Discussion:** We demonstrate a novel and translational method wherein β-NGF can be encapsulated within PEGDMA microrods for local delivery and that β-NGF bioactivity is maintained resulting in improved bone fracture repair.

## Introduction

Bone fractures are one of the most common injuries worldwide, with an estimated 178 million new fractures a year and 445 million prevalent fractures (Wu et al., 2021). Although bones possess the ability to fully regenerate, fractures negatively impact the quality of life of patients throughout the time course of healing, which can take 3–6 months under normal conditions (Bonafede et al., 2013). Delayed healing or failure to heal (non-unions) occur in 5%–10% of cases leading to healing times often exceeding 9 months and/or requiring multiple surgeries to achieve union (Marsell and Einhorn, 2010; Ekegren et al., 2018). Co-morbidities such as age, smoking, diabetes, and obesity drastically increase the prevalence of impaired bone healing to upward of 50% of fracture patients (Dickson et al., 1995; Penn-Barwell et al., 2013). Currently, surgical application of autologous bone grafts or adjustments to the orthopaedic hardware are the standard of care approaches to stimulate healing. Surgical intervention is associated with significant challenges including donor site morbidity, longer recovery time, increased costs, and risk of infection, among others (Khan et al., 2005; Roberts and Rosenbaum, 2012). There are no pharmacologic reagents approved to accelerate fracture healing or prevent non-union (Kostenuik and Mirza, 2017). Given these limitations with surgical methods, there is an unmet clinical need to develop pharmacological approaches to improve bone regeneration without surgery.

Bone morphogenetic proteins (BMPs) are perhaps the most established osteoanabolic growth factors and have been used for regenerating bone through a direct effect on osteoblastic differentiation of osteochondral progenitors (Lukač et al., 2020). Of the BMPs, BMP-2 is the only one with FDA approval for application in problematic fractures; specifically, BMP-2 is indicated for surgical application on a sponge scaffold in open tibial shaft fractures stabilized with an intramedullary nail within 14-days of the initial fracture (US Food and Drug Administration Public Health Notification, 2004; McKay et al., 2007). Currently, clinical application of BMP-2 in fracture repair remains limited due to its uncertain efficacy, high cost, and growing list of adverse outcomes that includes inflammatory complications, ectopic bone formation, and osteolysis (US Food and Drug Administration Public Health Notification, 2008; James et al., 2016). The serious complications are believed to be driven by the supraphysiological doses needed to achieve therapeutic efficacy and lack of drug delivery approaches to mitigate side effects. Thus, it is prudent to explore novel osteobiologics to accelerate fracture repair and to develop drug delivery systems designed to localize the effect and mitigate adverse outcomes.

While therapeutic use of BMPs have focused on promoting direct bone formation [intramembranous ossification (Kostenuik and Mirza, 2017)], bone fractures heal primarily through endochondral ossification. Endochondral ossification, or indirect bone formation, occurs when an avascular, aneural cartilage intermediate converts into a vascularized and innervated bone. Endochondral repair is a dynamic process that proceeds through four overlapping steps: 1) formation of hematoma at the fracture site and activation of the pro-inflammatory phase, 2) formation of a fibrocartilaginous callus within the bone gap, 3) hypertrophy, mineralization, and transformation of the cartilage to form trabecular bone, and 4) remodeling and compact bone formation (Bahney et al., 2015; Kostenuik and Mirza, 2017; Wong et al., 2018; Bahney et al., 2019). The disconnect between current therapies and the endogenous mechanism of fracture repair represents a potential explanation for poor or inconsistent outcomes with existing osteoinductive therapeutics.

Nerve growth factor (NGF) is a potent regenerative factor established for its role in regulating differentiation, growth, survival, and plasticity of cholinergic neurons (Levi-Montalcini, 2004; Niewiadomska et al., 2011). Interestingly, recent studies have also found NGF is critical in long bone development and repair, suggesting it could be a novel osteoinductive treatment for bone fractures. NGF signaling via its high-affinity receptor, tropomyosin receptor kinase A (TrkA), was first shown to modulate long bone development and skeletal adaptations to mechanical loads (Tomlinson et al., 2016; Tomlinson et al., 2017). More recently the role of NGF in bone repair has been explored. Following stress fractures, which heal through intramembranous ossification, NGF/TrkA signaling triggers reinnervation, vascularization, and osteoblastic activity during repair (Li et al., 2019). In parallel, our group has detailed the temporal expression of NGF and TrkA during long bone fracture repair and identified endogenous expression peaks in the cartilaginous phase of healing and that timing exogenous injections of recombinant beta NGF (β-NGF) with this peak resulted in accelerated endochondral fracture repair (Rivera et al., 2020). Mechanistically we found β-NGF delivery promoted cartilage to bone transformation by upregulating the Wingless/Integrated (Wnt) and Indian Hedgehog (Ihh) pathways (Rivera et al., 2020). However, as our published work demonstrated, drug administration by local injection required high dosages and repeated administration to elicit therapeutic effects (Rivera et al., 2020). Such dosing regimens will likely reduce patient compliance. In addition, the inherent limitations of soluble β-NGF delivery including its rapid inactivation by physiological conditions and lack of control over local concentrations creates an opportunity to develop controlled delivery platforms that can achieve sustained drug delivery in the absence of surgical implantation (Langer, 1998; Alastra et al., 2021; Hoare and Kohane, 2008).

Localized drug delivery to the fracture site with the use of injectable microparticles made of polymeric hydrogels is a highly translatable strategy to overcome the limitations associated with oral administration or surgical implantation. Poly (ethylene glycol) (PEG)-based hydrogels are clinically relevant polymer systems based on their low-immunogenic, non-cytotoxic, and biocompatible properties (D’souza and Shegokar, 2016). As such, PEG has had extensive biomedical uses for years, particularly in drug delivery. Modifying PEG hydrogels with methacrylation (PEGDMA) enables these monomers to be easily formed into three-dimensional (3D) polymers with defined shapes and size through initiation of free radical chain photopolymerization of the methacrylate groups at each end of the PEG chain (Choi et al., 2019). Proteins can be readily loaded into PEGDMA microparticles to allow for localized and sustained delivery with release kinetics tuned by altering the crosslinking density of the polymer (Li and Mooney, 2016). Previously, our group has fabricated high aspect ratio PEGDMA microrods by photolithography for targeted drug delivery (Doroudian et al., 2014; Peña et al., 2015). High-aspect ratio microarchitectures, such as microrods, create a high surface area to volume ratio and have been proven to evade internalization by phagocytic cells such as macrophages (Champion and Mitragotri, 2006; Champion and Mitragotri, 2009) thereby decreasing the local inflammatory response and favoring a regenerative response over a fibrotic response (Ayala et al., 2010).

In this work, we build on our foundational research showing recombinant β-NGF promotes endochondral repair in a murine fracture model (Rivera et al., 2020) by encapsulating the protein in PEGDMA microrods for delivery to the fracture site. We hypothesized that localized delivery of β-NGF from microrods will promote enhanced endochondral fracture repair relative to soluble β-NGF and untreated controls. Using a high throughput approach to fabricate PEGDMA, we varied the concentration of PEGDMA precursor solution (v/v) to evaluate its effects on protein loading and release. We found that changing the polymer network density did not affect the initial loading of β-NGF but altered the release and retention of β-NGF over 7 days through distinct physiochemical interactions. To confirm β-NGF encapsulated within these microrods maintains bioactivity, we utilized *in vitro* studies with erythroleukemia cells, a TrkA expressing cell line, to demonstrate retained cellular growth relative to other treatment groups. Subsequently, we established that PEGDMA microrods can be delivered and retained adjacent to the fracture callus for at least 7 days post-injection. Efficacy studies using our established murine model demonstrate that the mice receiving β-NGF loaded PEGDMA microrods exhibited improved fracture repair relative to soluble β-NGF or negative control. Herein, we describe an injectable system for localized β-NGF delivery for accelerating endochondral fracture repair.

## Methods

### PEGDMA microrod fabrication

Microrods were fabricated as previously described (Ayala et al., 2010). Briefly, 2, 2-dimethoxy-2-phenylacetophenone (DMPA) was dissolved in 1-vinyl-2-pyrrolidone at a concentration of 100 mg/mL and added at a 1:10 v/v ratio to poly (ethylene glycol) dimethacrylate (PEGDMA, molecular weight 750 kDa) dissolved in phosphate-buffered saline (PBS). 75% and 90% PEGDMA microrods were created by varying the % v/v PEGDMA to PBS. Photolithography was used to create microrods designed to have the dimensions of 100 × 15 × 15 μm. Silicon wafers were pre-cleaned with oxygen plasma and coated with a 15 μm thick layer of PEGDMA solution. These wafers were subsequently exposed to a 365 nm light source using a Karl Suss MJB3 or OAI 200 mask aligner. Microrods on the wafer were rinsed and removed with 70% ethanol while gently scraping with a cell scraper. The collected microrods were centrifuged and rinsed in 70% ethanol then washed three times in 0.05% Tween-20 and PBS before being resuspended in sterile deionized water (diH_2_O) with 10% sucrose and 0.05% tween-20 to prevent aggregation. Aliquots of ∼100,000 PEGDMA microrods were then lyophilized, sealed, and stored at 4°C until further use. A subset of PEGDMA microrods was resuspended in PBS and micrographed under bright field (BF). Another subset was stained with the low molecular weight dye 4′, 6-diamidino-2-phenylindole (DAPI) 1 μg/mL in PBS for 5 min, washed with PBS gently three times, then immediately imaged using a Nikon Ti microscope.

### β-NGF elution from PEGDMA microrods

75 (medium) and 90% (v/v) (high) PEGDMA microrods (∼100,000/aliquot) were resuspended in 20 μL of 1 mg/mL (20,000 ng in 20 μL) recombinant human β-NGF (Peprotech). After resuspension, the microrods were allowed to passively absorb protein for 30 h in 4°C with gentle shaking. After loading, microrods were washed thrice in diH_2_O, gently spun down to pellet in tube, and the supernatants collected and stored at −80°C until further use. Washed β-NGF loaded PEGDMA microrods were further divided into samples consisting of 16,000 microrods/microtube and suspended in 250 μL of PBS (pH 7.4) containing 0.05% Tween-20 (PBS-Tween). Samples were placed on an orbital shaker (100 RPM) in an incubator set at 37°C. To examine the release profile of β-NGF from PEGDMA microrods, microrods were gently spun down, 250 μL of PBS-Tween was removed, and then fresh 250 μL of PBS-Tween was added. This process of collection and replenishing was done at 2, 4, 6, 8, 17, 24, 48, 72, 96, 120, 144, and 168 h for each PEGDMA group and done in triplicate. Collected supernatants at each designated timepoint were immediately flash frozen and stored at −80°C until further use. ELISAs for human β-NGF (RayBiotech) were performed per the manufacturer’s instructions and both loading efficiency and hourly release amounts were calculated by an established standard curve. To determine the loading efficiency in 100,000 microrods, the following equation was used:

$$Loading\;efficiency\;\%=\left(\frac{X_{i}-X_{t}}{X_{i}}\right)\;*\;100$$ (4)

Where X_i_ is the quantity of drug added initially during loading and X_t_ is amount of protein in the supernatant after 30 h. Loading of PEGDMA microrods with β-NGF was done similarly in subsequent assays detailed below.

### PEGDMA microrod mesh size calculation

To better understand the transport of β-NGF through our microrods, we performed swelling studies with microrods. We weighed microrods in diH2O to determine the swollen mass, and subsequently lyophilized samples to determine the dry weight of the hydrogels. From these measurements, we calculated the mass swelling ratio (*Q*_*M*_), and subsequently determined the volumetric swelling ratio QV (Marsano et al., 2000) (Eq. 1).

$$Q_{v}=1+\frac{\rho_{polymer}}{\rho_{solvent}}\left(QM-1\right)$$ (1)

Then, using the modified Flory-Rehner theory for hydrogels prepared in the presence of water (Peppas et al., 2006), we calculated the number average molecular weight between crosslinks within the polymer network (*M*_*C*_) (Eq. 2).

$$\frac{1}{M_{C}}=\frac{2}{M_{n}}-\frac{\left(\frac{\nu }{V_{1}}\right)[\ln\left(1-\nu_{2,s}\right)+\nu_{2,s}+\chi \nu_{2,s}^{2}}{\nu_{2,r}\left[{\left(\frac{\nu_{2,s}}{\nu_{2,r}}\right)}^{\frac{1}{3}}-\left(\frac{\nu_{2,s}}{\nu_{2,r}}\right)\right]}$$ (2)

Where *M*_*n*_ is the number average molecular weight of the polymer, *ν* is the specific volume of the dry polymer, *V*_*1*_ is the molar volume of the solvent, χ is the Flory polymer solvent interaction parameter, *ν*_*2,s*_ is the polymer volume fraction in the swollen state (defined as $\frac{1}{Q_{V}}$ ) (Meenach et al., 2010), and *ν*_*2,r*_ is the polymer volume fraction in the relaxed state after crosslinking but before swelling.

The mesh size (**ξ**) of PEGDMA microrod hydrogels was then calculated using the universal Canal-Peppas Equation described by Richbourg and Peppas (Richbourg and Peppas, 2020) (Eq. 3).

$$\xi =\varphi^{-\frac{1}{3}}{\left(\left(1-\frac{2}{f}\right)\iota^{-2}C_{\infty }\frac{\lambda M_{c}}{M_{r}}\right)}^{\frac{1}{2}}$$ (3)

Where *φ* is the general polymer volume fraction, *f* is the junction functionality in the polymer network, ι is the weighted average of bond lengths per repeating unit, $C_{\infty }$ is the polymer-sepcific characteristic ratio for a long chain, *λ* is the polymer backbone bond factor, and *M*_*r*_ is the molecular weight of the polymer repeat unit.

### β-NGF and PEGDMA microrod physiochemical interactions

To assess the physiochemical interactions between β-NGF and PEGDMA microrods, 1 M Urea (Sigma-Aldrich) and 1 M sodium chloride (NaCl, Sigma-Aldrich) were used as a chaotropic and salting-out agents, respectively. PEGDMA microrods were prepared, lyophilized, loaded with β-NGF, and aliquoted at 16,000 microrods/microtube as detailed in the *PEGDMA microrod fabrication* and *β-NGF elution from PEGDMA microrods* section. To extract β-NGF after loading, the aliquoted β-NGF-microrods were immediately resuspended in either 250 μL of PBS-Tween, 1 M Urea or 1 M NaCl for 2 h. After 2 h of release, the microrods were gently spun down to pellet in tube, and the supernatants collected and stored at −80°C until further use. To assess the retained β-NGF after *in vitro* release, the β-NGF microrods elution assay was repeated with replacement of 250 μL PBS-Tween at 2, 4, 6, 8, 17, 24, 48, 72, 96, 120, 144, and 168 h and afterwards were gently pelleted and resuspened in either 1 M Urea or 1 M NaCl for 2 h. After 2 h of release, the microrods were gently spun down to pellet in tube, and the supernatants collected and stored at −80°C until further use. ELISAs for human β-NGF (RayBiotech) were performed per the manufacturer’s instructions and protein extracted from PEGDMA microrods after loading and *in vitro* release was calculated by an established standard curve.

### Attenuated total reflectance fourier transform infrared spectroscopy (ATR-FTIR)

For ATR-FTIR, high PEGDMA microrods were prepared, lyophilized, and loaded with β-NGF as detailed in the *PEGDMA microrod fabrication* and *β-NGF elution from PEGDMA microrods section*. After loading, the β-NGF loaded microrods were gently washed and resuspended in water for lyophilization. For non-loaded PEGDMA microrods, the microrods were prepared exactly as the loaded microrods but without the addition of β-NGF during the loading process. To examine the β-NGF loaded PEGDMA microrods after 168 h of release, β-NGF loaded PEGDMA microrods were aliquoted at 16,000 microrods/microtube and elution experiments were performed as described in the *β-NGF elution from PEGDMA microrods* section. After the elution experiments, the β-NGF loaded PEGDMA microrods were gently washed and resuspended in water for lyophilization. All samples were stored at −80°C until ATR-FTIR analysis. ATR FTIR (Spotlight 400) was performed with a scan resolution of 8 cm^−1^ from 4,500 to 450 cm^−1^ and a constant anvil pressure value of 45. Using the Spectrum software (Perkin Elmer), a background scan was recorded immediately after each sample scan to facilitate background correction. After background corrections, an ATR correction algorithm was applied, and the data was normalized to the highest ordinate value.

### Erythroblast (TF-1) cell number and viability

TF-1 cell number and viability assay was modified from the established method with modifications (Malerba et al., 2015). Briefly, TF-1 cells (ATCC) were cultured for 7 days in RPMI 1640 Medium modified with 2 mM L-glutamine, 10 mM HEPES, 1 mM sodium pyruvate, 4,500 mg/L glucose, and 1,500 mg/L sodium bicarbonate (ATCC) supplemented with 2 ng/mL recombinant human Granulocyte-Macrophage Colony-Stimulating Factor (Sigma-Aldrich) and 10% fetal bovine serum (hereafter called full media). While cells were growing, 100,000 high concentration (90% w/v) PEGDMA microrods were loaded with β-NGF and aliquoted at 16,000 as described in the *β-NGF elution from PEGDMA microrods* method and then resuspended in 250 μL of full media. β-NGF-loaded microrods were kept in full media for 168 h and then the gently spun down and 250 μL removed and stored at −80°C. A set of non-loaded microrods were treated exactly like the β-NGF-loaded microrods and served as a control. As an additional control, the soluble β-NGF was incubated in media for the same duration. Following 7 days of cell growth, confluent TF-1 cells were collected by centrifugation and resuspended into 96-well microplates with 2,000 cells per well containing 40 µL of complete RPMI1640. 60 min after replating, cells were treated with 250 μL of full media, β-NGF conditioned media from microrods, non-loaded microrod conditioned media, or 2,000 ng of soluble β-NGF. Cells were cultured in these conditions for 40 h prior to assessing cell viability and number via a PrestoBlue viability assay (Invitrogen) per the manufacturers protocol. Briefly, the 10X reagent was warmed to room temperature and then 10 μL was added to each well for every 90 µL. The reduction of resazurin by cells was allowed to proceed for 60 min prior to centrifuging the 96-well microplate (1,000 RPM for 5 min) and measuring fluorescence on a plate reader at 560/590 (emission/excitation). A standard curve of known TF-1 cell number was done in parallel to quantify the total number of cells in each well. The treatment groups and controls were all done in triplicate. Data is represented as a fold change relative to the full media treated only control group.

### Murine tibial fracture with localized drug delivery

Approval was obtained from the University of California, San Francisco (UCSF) Institutional Animal Care and Use Committee (IACUC) prior to performing the mouse studies and the procedures were carried out in accordance with approved guidelines and regulations. Studies were conducted on the C57BL6/J wild type strain obtained from Jackson Labs. Briefly, adult (10–16 weeks) male mice were anesthetized via inhalant isoflurane, and closed non-stable fractures were made mid-diaphysis of the tibia via three-point bending fracture device (Bahney et al., 2014). Fractures were not stabilized as this method promotes robust endochondral repair. After fractures were created, animals were provided with post-operative analgesics (buprenorphine sustained release). Animals were socially housed and allowed to ambulate freely.

Percutaneous injections into tibial fracture calluses of mice were administered 7 days post-fracture. A precise microliter Hamilton© syringe was utilized for all injections wherein experimental agents were injected in 20 μL of PBS. Experimental groups are as follows: Controls injected with sterile PBS, β-NGF group injected with 2000 ng of β-NGF in PBS, non-loaded microrods group injected with 16,000 PEGDMA microrods in PBS, and β-NGF microrods group injected with 16,000 PEGDMA microrods loaded with 2000 ng of β-NGF.

### Micro-computed tomography (μCT)

μCT analysis was performed to quantify the volume and quality of the new bone as previously described (Hu et al., 2017a; Cheng et al., 2020). Fracture tibias were dissected free of attached muscle 14 days post-fracture, fixed in 4% PFA and stored in 70% ethanol. Fracture calluses were analyzed in the UCSF Core Center for Musculoskeletal Biology (CCMBM, NIH P30 funded core) using the Scanco μCT50 scanner (Scanco Medical AG, Basserdorf, Switzerland) with 10 μm voxel size and X-ray energies of 55 kVp and 109 μA. A lower excluding threshold of 400 mg hydroxyapatite (HA)/mm^3^ was applied to segment total mineralized bone matrix from soft tissue in studies of control and treated mice. Linear attenuation was calibrated using a Scanco hydroxyapatite phantom. The regions of interest (ROI) included the entire callus without existing cortical clearly distinguished by its anatomical location and much higher mineral density. μCT reconstruction and quantitative analyses were performed to obtain the following structural parameters: volume fraction (bone volume/total volume as %), trabecular connective density as trabecular bifurcations (#/mm^3^), and bone mineral density (mg HA/cm^3^).

### Histology and histomorphometry

Tibias were harvested 12, 14-, and 21-days post-fracture (5, 7, or 14 days post-injection of microrods) to observe microrod localization. At time of collection, tibias were fixed in 4% PFA and decalcified in 19% EDTA for 14 days at 4°C with rocking and solution changes every other day. Tibias were processed for paraffin embedding, serial sections were cut at 10 μm (3 sections per slide), and Hall-Brundt’s Quadruple (HBQ) staining protocol was done to visualize the bone (red) and cartilage (blue) as previously described to localize PEGDMA microrods (Hu et al., 2017; Rivera et al., 2020). The sections were mounted on slides with Permount™ mounting medium and brightfield images were captured on a Leica DMRB microscope.

Fracture callus composition and inflammatory response was determined using quantitative histomorphometry of tibia harvested 14 days post-fracture. Standard principles of histomorphometric analysis (Hu et al., 2017) were utilized to quantify the bone and cartilage fraction in the fracture callus using the first section from every 10th slide analyzed, such that sections were 300 μm apart. Images were captured using a Nikon Eclipse Ni-U microscope with Nikon NIS Basic Research Elements Software version 4.30. Quantification of callus composition (cartilage, bone, fibrous tissue, background) was determined using the Trainable Weka Segmentation add-on in Fiji ImageJ (version 1.51.23; NIH, Maryland, USA) (Malhan et al., 2018). Volume of specific tissue types was determined in reference to the entire fracture callus by summing the individual compositions relative to the whole.

### Statistical analysis

Individual dots on graphs represent biological replicates, error bars represent standard deviation (SD). Measurements were taken from distinct samples. Data were analyzed using GraphPad Prism (version 8, GraphPad Software, San Diego, CA). For release data, a repeated measures two-way ANOVA was performed followed by a Tukey’s HSD *post hoc* comparison test to determine statistical difference between independent groups at each respective timepoint. For all other data, a One-way ANOVA was used to determine statistical differences between multiple groups followed by Tukey’s HSD *post hoc* comparison testing. Significant differences were defined at *p* < 0.05.

## Results

### Injectable PEGDMA microrod fabrication via photolithography

**FIGURE 1:**
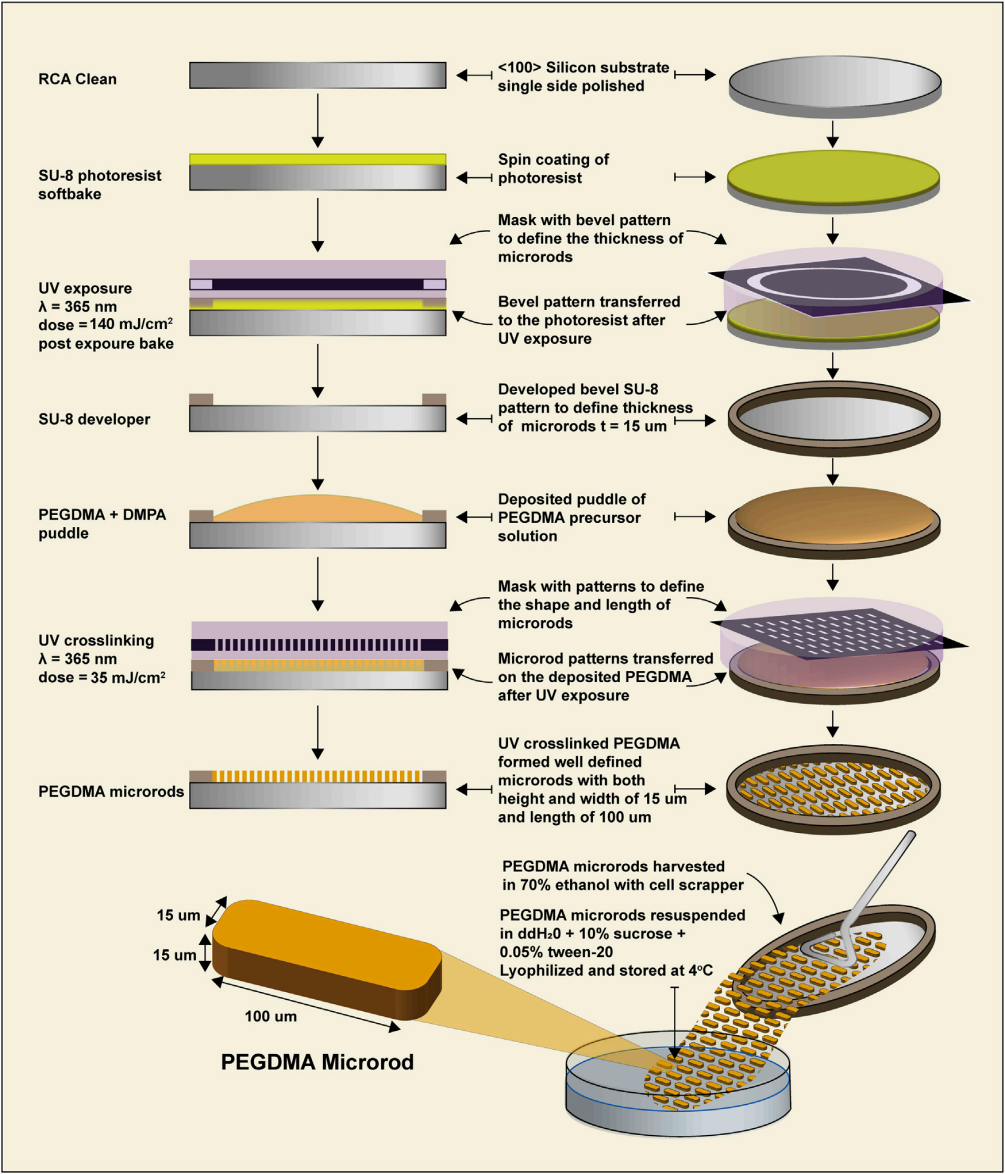
Schematic of PEGDMA microrod fabrication via photolithography.

PEGDMA microrods were fabricated through a process of photolithography (Figure 1). The exact dimensions of the PEGDMA microrods can be carefully controlled by the photomask (length and width). The microrod height is determined by distance between the silicon wafer and the photomask which is manually controlled by use of the mask aligner. PEGDMA microrods are then cross-linked with UV irradiation, detached with a cell scraper from the silicon wafer and collected. Each individual PEGDMA microrod had the following dimensions: *height =* 15 μm, *width* = 15 μm, and *length* = 100 μm. The 3D structure of the microrods is formed through free radical chain photopolymerization of the methacrylate groups at each end of each 750 MW PEG monomer unit. This system provides a high-throughput method to produce highly uniform PEGDMA microrods.

### PEGDMA microrod macromer concentration influences protein retention

**FIGURE 2:**
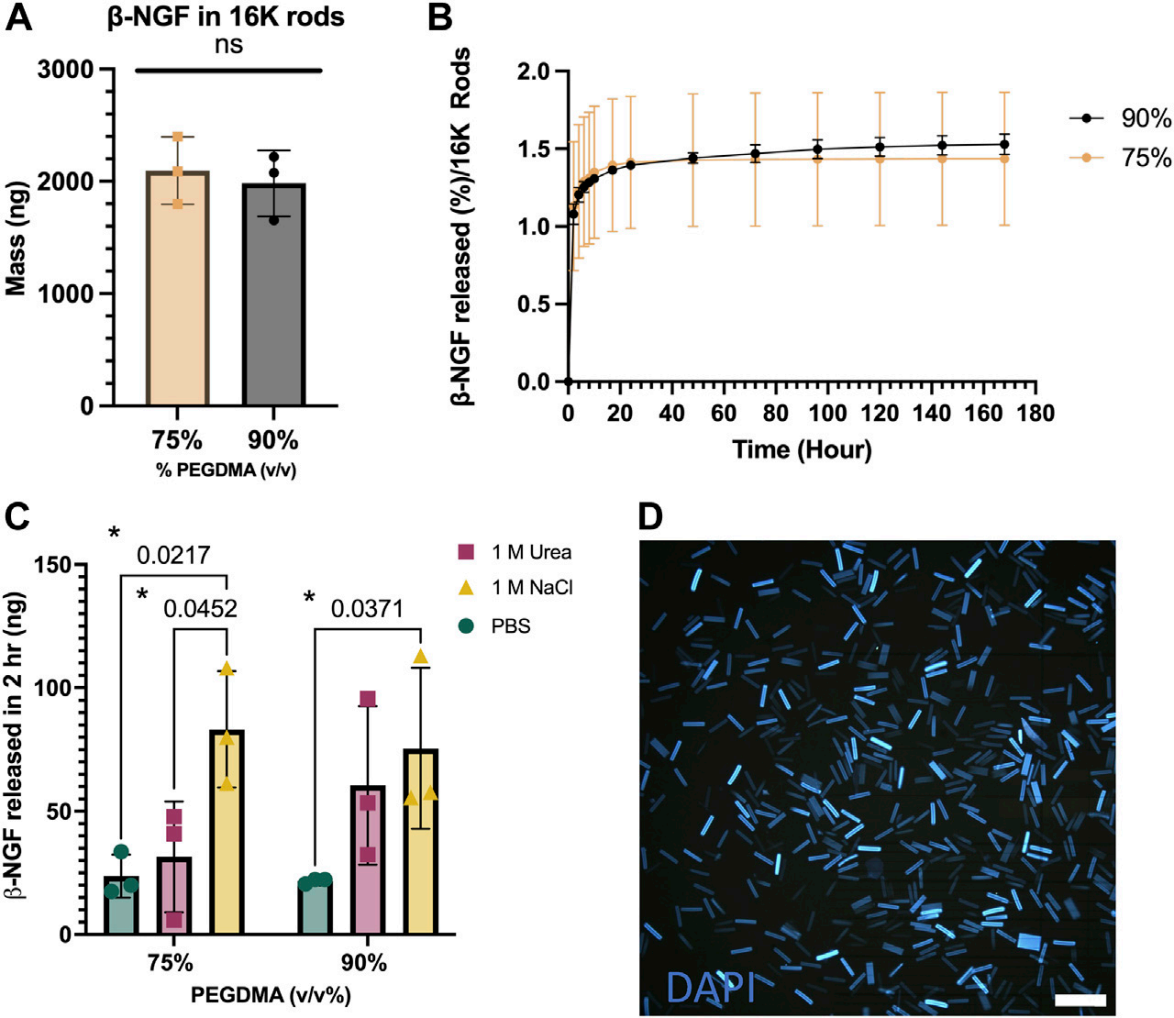
PEGDMA microrods are readily protein loaded. **(A)** Mass in 16,000 (16K) microrods. **(B)** Percent release of β-NGF from 16K 75 and 90% (v/v) PEGDMA microrods (*n* = 3). **(C)** β-NGF released (in ng) from 75 and 90% microrods after treatment with PBS, 1M NaCl, or 1 M Urea for 2 h. *p < 0.05 determined by Two-way ANOVA with Tukey’s post hoc test for multiple comparisons (*n* = 3). **(D)** DAPI-loaded 90% PEGDMA microrods, scale bar = 250 µm.

First, we aimed to examine how PEGDMA microrod polymer network density affected protein loading efficiency and subsequent release of β-NGF. To do this, the PEGDMA macromer was adjusted prior to photolithography to have a 75% or 90% concentration (v/v) (Figure 2A). After fabrication, the PEGDMA microrods were lyophilized to remove any residual liquid remaining and then incubated with 20,000 ng β-NGF (26 kDa) through a 30 h absorption step. Macromer concentration did not significantly affect loading efficiency with 75% and 90% v/v macromer concentrations exhibiting 65% and 61% loading efficiency, respectively (Supplementary Figure S1). Although over 2000 ng of β-NGF was loaded in 16,000 microrods for both macromer concentrations (Figure 2A), less than 2% of the total protein was released over the course of 168 h (Figure 2B). Moreover, the release profiles were not statistically different. These findings suggest that the PEGDMA microrods retain protein within the polymer mesh network. To assess retention within the microrods, a salting out approach was used to destabilize the protein within the microrods. After incubation with a high salt concentration for 2 h post-loading, a nearly 4-fold difference in elution was observed from both 75% and 90% microrods which was statistically higher than the respective microrods eluted in PBS (Figure 2C). In addition, due to the high presence of etheric groups within the PEGDMA polymer network, we aimed to further assess the potential contribution of hydrogen bonding. To test this, a common chaotropic agent, urea, was used to disrupt hydrogen bond interactions. Interestingly, after incubation in the presence of the chaotropic agent, additional β-NGF release was detected from both the 75% and 90% PEGDMA microrods with 2-fold more from the 90% PEGDMA microrods (Figure 2C). Given the higher macromer concentration (i.e., increased number of etheric groups) of 90% v/v PEGMDA microrods, additional ATR-FTIR analysis and theoretical calculations of these microrods were performed to assess spectral shifts of pre- and post-loaded microrods and the hydrogel mesh size, respectively. After loading of β-NGF, the PEGDMA macromer ester peak C = O at ∼1720 cm^−1^ underwent a subtle hypsochromic shift to ∼1723 cm^−1^, which might be due to weakening of the polymer intermolecular reaction and/or longer distance between the polymers main chain by interactions with β-NGF (Supplementary Figure S1). In addition, the shift to ∼1723 cm^−1^ was still observed after *in vitro* release indicating that β-NGF was still present within the PEGDMA microrod (Supplementary Figure S1). Furthermore, the encapsulation of β-NGF within the 90% PEGDMA microrod was confirmed through theoretical calculation of the mesh size (ξ), which was determined to be ∼1.865 nm compared to the hydrodynamic radius of β-NGF which has been reported to be between 2–4 nm (Stroh et al., 2003; Conovaloff et al., 2011).

Given the high retention β-NGF over the course of 168 h, we aimed to see if additional β-NGF could be extracted from the polymer matrix after this time frame. We repeated the release assay for 168 h with PBS-Tween, but at the final collection, the 75% and 90% PEGDMA microrods were incubated with either 1 M Urea or 1 M NaCl (Supplementary Figure S1). For both PEGDMA microrod concentrations, a minimal amount (∼20 ng) was detected after 1 M Urea treatment. However, nearly half the amount of the initially loaded β-NGF was released from 70% PEGDMA microrods after incubating the rods with 1 M NaCl.

We then wanted to examine the aggregation behavior of microrods, as this could adversely affect *in vivo* injections. DAPI, a commonly used nuclear counter stain, can be easily absorbed into the high PEGDMA microrods and visualized with fluorescent microscopy. As shown in Figure 2C, the DAPI-stained microrods are uniform in size and do not exhibit any aggregation, indicating good dispersity in solutions.

### β-NGF bioactivity retention and elevated cell number after treatment with PEGMDA microrods

**FIGURE 3:**
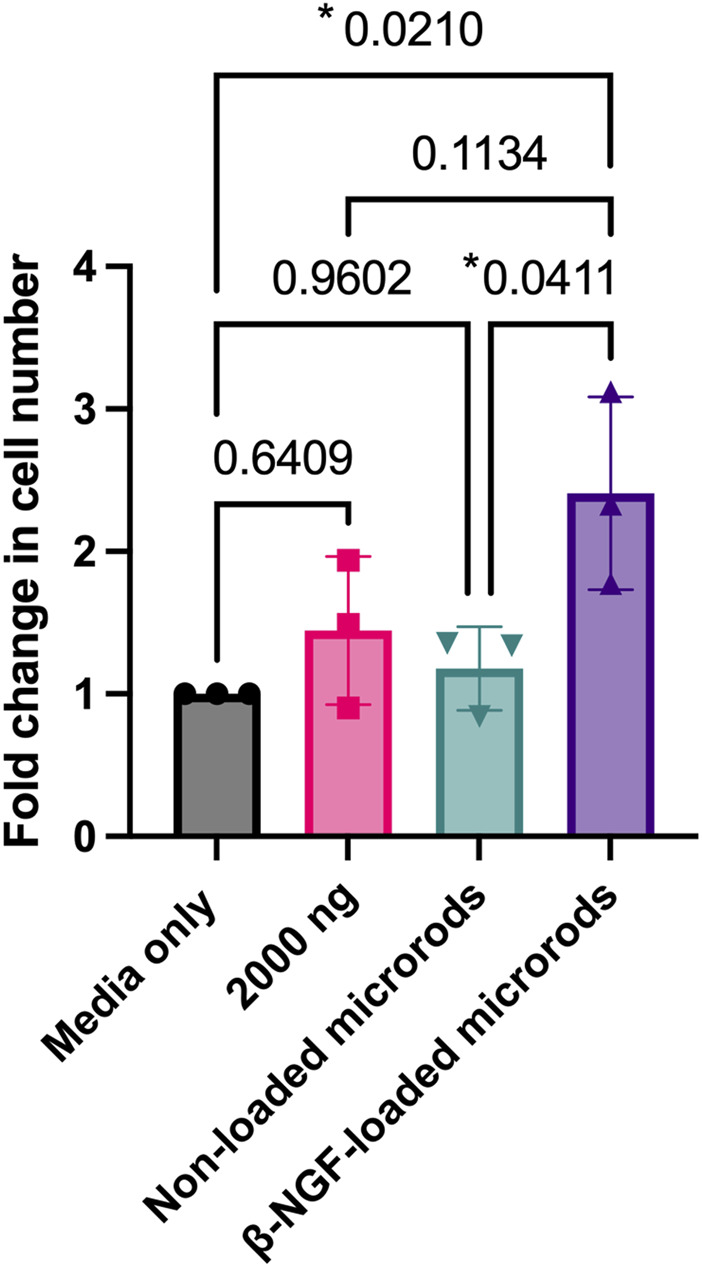
β-NGF loaded into PEGDMA microrods retain bioactivity and increased cell number. PEGDMA microrods were incubated in cell media for 168 hours and the media was collected and used to treat the cells for 40 hours. Fold change in TF-1 cell number for each experimental group is relative to the media only treated cells. Error bars represent SD, *p < 0.05 determined by ANOVA with Tukey’s post hoc test for multiple comparisons (*n* = 3).

We next tested whether β-NGF retained bioactivity after loading within the PEGDMA microrods. To do this, we measured the cellular response of the erythroleukemia TrkA expressing cell line, TF-1, 40 h following treatment with media that was conditioned with PEGDMA microrods loaded with 2000 ng β-NGF for 168 h. TF-1 cells are well established to proliferate in response to β-NGF (Malerba et al., 2015; Recombinant Human β-NGF, 2020). As controls, TF-1 cells were cultured with non-conditioned media, non-loaded microrod conditioned media, or media containing 2000 ng of soluble β-NGF. Treating the cells with 2000 ng soluble of β-NGF resulted in cellular growth, and we observed a 1.5-fold increase in cell number relative to the media only control (Figure 3). No significant cellular response was observed in response to culture with the non-loaded PEGDMA microrods, indicating no positive or negative effects of any hydrogel byproduct. Following culture with the β-NGF loaded microrods, cell number was ∼2.5-fold increased relative to the media only control and 60% higher than the free β-NGF, suggesting retention in the PEGDMA microrods helped to stabilize the bioactivity of the protein. Taken together these data support that the microrods themselves do not adversely affect cell growth and that bioactivity of the β-NGF is enhanced when delivered from microrods relative to the same concentration of the soluble protein.

Based on these results, we aimed to validate therapeutic efficacy through *in vivo* analysis in a fracture model. Due to the ability of 90% v/v PEGDMA microrods to load and retain larger amounts of bioactive β-NGF within the polymer matrix compared to 70% microrods, as well as their dispersion in solution, 90% microrod were further used in subsequent *in vivo* testing.

### β-NGF loaded PEGDMA microrods promote endochondral bone formation

**FIGURE 4:**
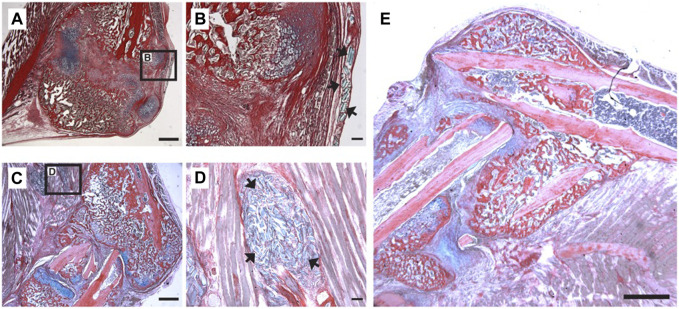
Localization of PEGDMA microrods within tibial fracture calluses. Representative micrographs of **(A)** low and **(B)** high magnification of HBQ-stained fracture calluses 5 days post-microrod injection. Representative micrographs of **(C)** low and **(D)** high magnification of fracture calluses 7 days postmicrorod injection. Arrows indicate PEGDMA microrods within calluses. **(A,C)** Scale bars = 1 mm, **(B,D)** Scale bars = 100 µm. **(E)** Representative micrograph of fracture callus 14 days post-injection, scale bar = 1 mm.

Our data thus far has demonstrated that PEGDMA microrods can be loaded with β-NGF, mesh network density can be tuned to minimize physiochemical effects on protein loss from the microrod, and the subsequent protein bioactivity is maintained after 7-days of retention within the microrod. Given that protein loading and stability is maintained after 7-days, we aimed to test whether β-NGF loaded PEGDMA microrods could accelerate endochondral fracture repair. Consistent with our prior study, we utilized our established murine model of long bone healing wherein closed, mid-shaft fractures were created in the right tibia of adult wild type mice through three-point bending to promote robust endochondral repair (Bahney et al., 2014; Hu et al., 2017; Rivera et al., 2020) (Supplementary Figure S2). Previously, we demonstrated that β-NGF was most effective in promoting fracture repair when delivered 7 days post-injury, during the cartilaginous phase of bone healing (Rivera et al., 2020). Based on this we chose to similarly deliver PEGDMA microrods 7 days post-injury using a Hamilton syringe for percutaneous injection directly at the fracture site. First, we confirmed that the microrods could effectively be delivered to fracture callus by injecting 16,000 microrods suspended in 20 μL saline and harvesting the samples 5–14 days following delivery for histology (Figure 4). Histological sections of the fracture callus were stained with Hall-Brunt’s Quadruple (HBQ) staining to distinguish the cartilage (blue) and bone (red) tissues. Microrods (staining light blue) were evident adjacent to the fracture callus at days 5–7 post-injection (Figures 4A–C) but not found in any samples 14-days following injection (Figure 4D). Importantly we observed normal progression of healing without evidence of granulation tissue around the microrods indicating that the microrods do not negatively impact the healing response nor induce a clinically significant inflammatory response (Figures 4A–E).

**FIGURE 5:**
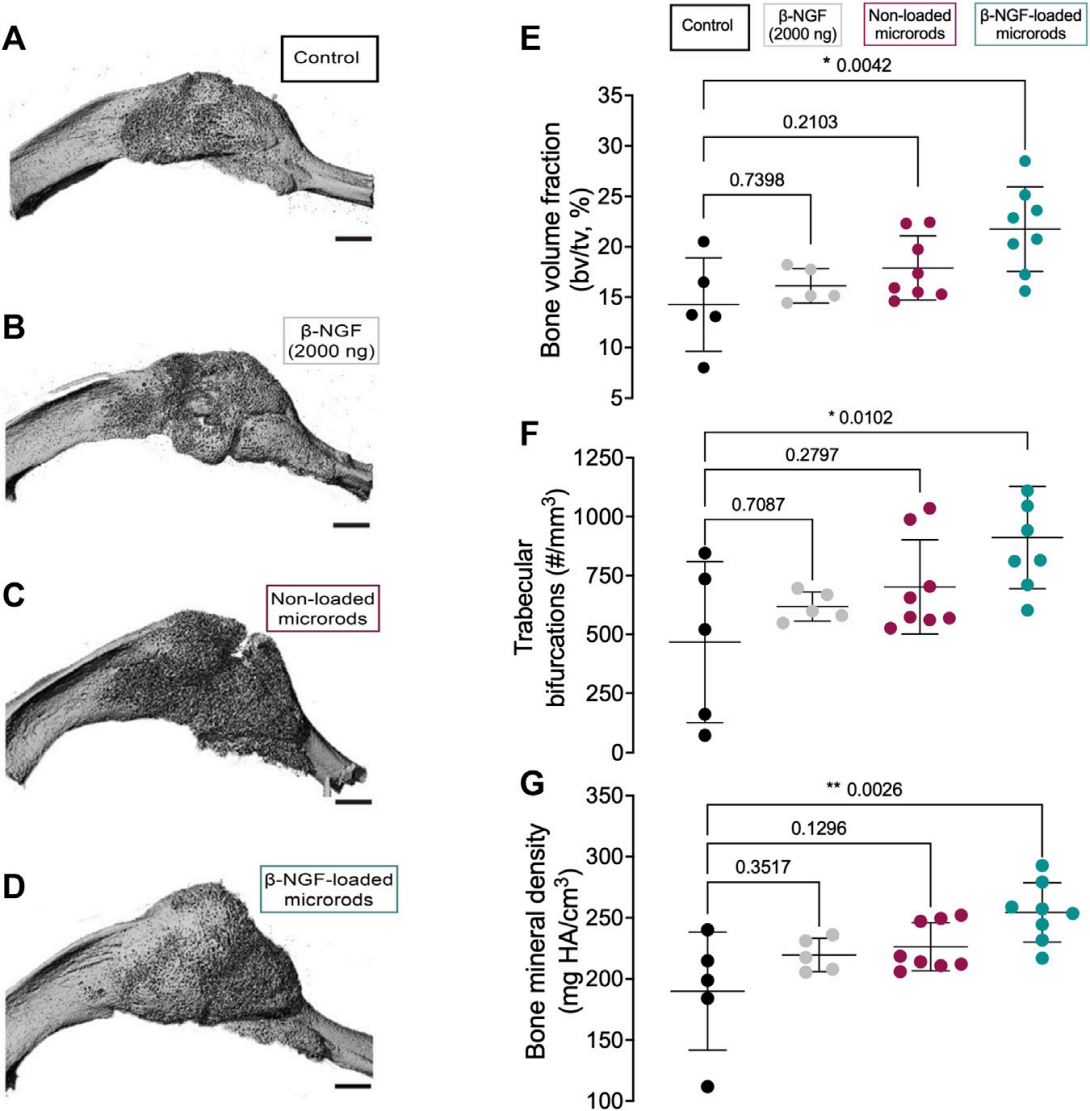
Micro-computed tomography (μCT) analysis of newly formed bone within fracture calluses. Representative three-dimensional images of tibial fracture calluses from mice treated 14 days post-fracture with **(A)** saline as controls **(B)** single dose of β-NGF (2000ng) **(C)** non-loaded PEGDMA microrods and **(D)** PEGDMA microrods loaded with β-NGF. Scale bars = 1 mm. Quantification of **(E)** bone volume fraction **(F)** trabecular connective density and **(G)** bone mineral density. Error bars represent SD, * *p* < 0.05, ** *p* < 0.01 determined by ANOVA with Tukey’s post hoc test for multiple comparisons.

We next evaluated the therapeutic efficacy β-NGF-loaded PEGDMA microrods compared to placebo injections (20 μL saline, negative control), a single dose of free β-NGF (2000 ng), and non-loaded PEGDMA microrods. All therapeutic groups were administered 7 days post-fracture by percutaneous injection and animals were allowed to heal for 7 days (14 days post-fracture) at which point the calluses were harvested for functional assessments of bone healing. Micro-computed tomography (μCT) images suggest that the β-NGF microrods group have the largest most consolidated bony callus compared to the other groups (Figures 5A–D). Quantification of the bone volume fraction (BVF) confirmed the highest BVF in the β-NGF microrods group with a significantly higher BVF (∼52% increase) compared to saline controls (Figure 5E). β-NGF microrods treatment also significantly increased trabecular bifurcations (TB, ∼95% increase) and bone mineral density (BMD, ∼34% increase) compared to saline controls (Figures 5F, G) indicating more mature fracture calluses. Interestingly, a single dose of the free β-NGF did not improve bone formation to the same extent as β-NGF microrods and was not statistically different from the saline controls. Although not statistically different, the non-loaded microrods exhibited higher BVF, TB, and BMD when compared to the soluble β-NGF and saline treated groups.

### β-NGF loaded PEGDMA microrods reduce cartilaginous tissue volume in the fracture callus

**FIGURE 6:**
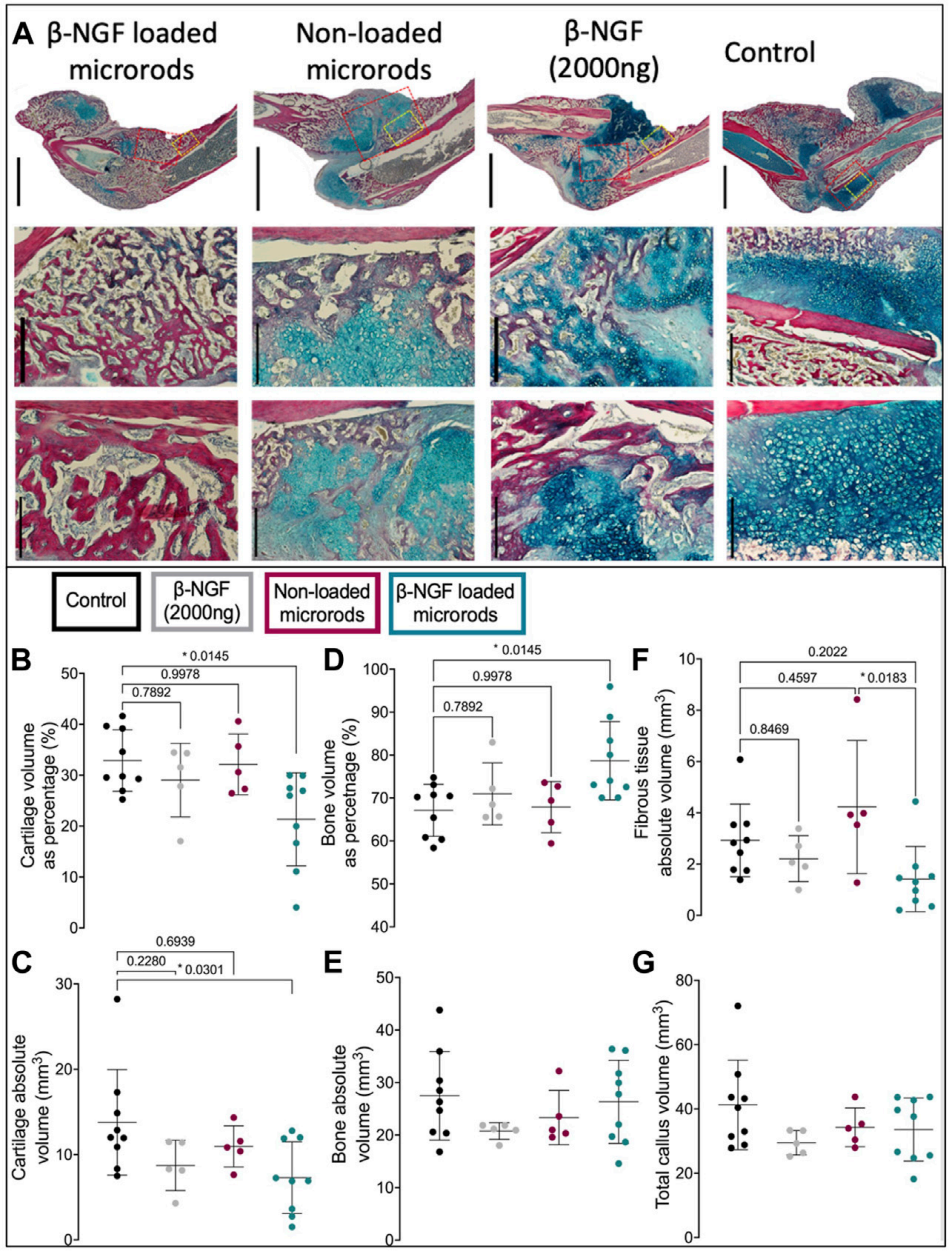
Single injections of PEGDMA microrods loaded with β-NGF promote endochondral bone repair. **(A)** Representative micrographs of HBQ-stained fracture calluses from mice 14 days post-fracture treated with saline as controls, single dose of soluble β-NGF (2000ng), non-loaded PEGDMA microrods, or PEGDMA microrods loaded with β-NGF. Upper row scale bars = 2mm, middle and lower row scale bars = 500 µm. Quantification of **(B)** cartilage percentage (cartilage volume/total volume), **(C)** absolute cartilage volume, **(D)** bone percentage (bone volume/total volume), **(E)** absolute bone volume, **(F)** fibrous percentage (fibrous volume/total volume), and **(G)** total callus volume. Error bars represent SD, *p < 0.05 determined by ANOVA with Tukey’s post hoc test for multiple comparisons.

To understand the differences noted by μCT at a more detailed tissue level, we utilized quantitative histology to differentiate the tissue composition within the fracture callus. The total volume of the fracture callus was not statistically different in any group (Figure 6G), and tissue compositions are provided as both a percentage of the total volume or the absolute volume of a specific tissue. HBQ-stained sections of legs harvested 14 days post fracture visually indicate that β-NGF microrods have the highest amount of new bone (red, Figure 6A). Saline treated fracture calluses had the highest volume of cartilage (blue) as both an absolute value and percentage of the total callus volume (32% ± 2%), with the least bone volume as percent composition (67% ± 2%) compared to other treatment groups indicating the least advanced healing (Figures 6B–E). Higher magnification histological images verify a large proportions of chondrocytes in the placebo injected fracture callus indicating that healing is just nearing the cartilage to bone transition phase in endochondral repair (Figure 6A). Near identical results were observed for the empty microrod treated fracture calluses with cartilage and bone volume at 32% ± 2.7% and 68% ± 2.7%, respectively (Figures 6B–E). A single injection of the soluble β-NGF treated fracture calluses resulted in slightly elevated levels of bone (71% ± 3.2%) and lowered cartilage volumes (29% ± 3.2%) relative to the empty microrods and untreated controls, but this effect was not statistically different than saline injections. β-NGF loaded microrods were the only treatment group to significantly change the fracture callus composition producing robust bone formation (79% ± 3%) (Figures 6D, E).

β-NGF microrod treated samples also show a significant visual reduction in cartilage and statistically different cartilage volume as both a percentage of the fracture callus (21% ± 3%) and absolute volume compared to saline controls (Figures 6B, C). Fibrotic tissue was also quantified to determine if there was a significant inflammatory response (Figure 6F). No group had statistically increased fibrotic tissue compared to the control group, although one animal in the non-loaded microrods group had a higher amount of fibrotic tissue resulting in larger variation from this group. β-NGF loaded microrods had the least amount of fibrotic tissue, statistically less than the non-loaded microrods, supporting that healing was the most advanced and that no adverse inflammatory response impeded proper tissue formation (Figures 6F,G).

## Discussion

Recent work in the field supports that NGF signaling plays a significant role in both intramembranous and endochondral ossification. The pioneering mechanistic studies first describe a critical role of NGF/TrkA signaling during skeletal development and adaptation to mechanical load (Tomlinson et al., 2016; Tomlinson et al., 2017). Subsequently our group delineated the spatiotemporal expression of TrkA during long bone fracture repair and identified the cartilaginous phase of healing as a key timepoint for exogenous β-NGF injections by promoting pathways associated with endochondral ossification (Rivera et al., 2020). Most recently, NGF/TrkA signaling was also shown to be acutely upregulated following stress fracture, triggering reinnervation, vascularization, and osteoblastic activity (Li et al., 2019). Stress fractures are a specific and unique subclass of fracture repair that heal through intramembranous bone formation. Together these studies suggest that NGF could be used as an osteoinductive drug to promote long bone fracture repair by synergistically promoting intramembranous and endochondral bone repair.

This study aimed to build on our previous work by developing a clinically relevant drug delivery system for β-NGF delivery using PEG-based hydrogel microparticles. In our previous study, we locally injected 500 ng of recombinant β-NGF once daily for 3 days to produce a quantifiable acceleration of fracture healing. However, β-NGF is a known hyperalgesic and preclinical studies utilizing murine models have demonstrated that hyperalgesia in mice can be experienced with daily injections of 100 ng and above (Hopkins and Slack, 1984; Shinoda et al., 2011). Thus, our goal is to balance the trophic benefit of β-NGF therapy while minimizing its hyperalgesic effects by providing sustained drug release at a dosing below this threshold. To avoid repeated doses, we aimed to use PEGDMA microrods as a clinically relevant drug delivery platform. The majority of PEGDMA microparticle delivery platforms use spherical particles for bone repair applications (Olabisi et al., 2010; Sonnet et al., 2013; Stukel et al., 2015). Uniquely, in our study we demonstrate the use of high aspect ratio microrods for fracture repair which may provide advantages compared to microspheres. For example, high aspect particles have been shown to evade phagocytosis or cellular internalization (Champion and Mitragotri, 2006; Champion and Mitragotri, 2009). This may allow for higher tissue residence time and thus extended local drug concentrations. Additionally, by employing photolithography, PEGDMA microrods can be controlled in terms of size and shape and produced in a high throughput fashion.

To tune hydrogel delivery systems as drug carriers, alterations to the cross-linking densities via changes in the macromer concentrations can be made to alter polymer mesh sizes, affecting subsequent drug loading and release (Li and Mooney, 2016). Several studies have demonstrated that increasing monomer concentrations can increase drug loading and improve release within hydrogel systems (Hoare and Kohane, 2008; Li and Mooney, 2016). Interestingly, we did not observe any statistically significant differences in the loading capacity of the microrods with different macromer concentrations. This is important because it demonstrates that although the mesh density is different, as we previously reported (Ayala et al., 2010), it does not impact the ability of the protein to be taken up by the network during the loading step. Moreover, both microrod concentrations exhibit high retention of β-NGF over 168 h *in vitro*, with only 2% of the total protein being released from the microrod mesh network in PBS-Tween. To elucidate potential β-NGF and PEGDMA interactions, we turned to a salting out approach commonly used for β-NGF column elution (Bocchini and Angeletti, 1969; Sakiyama-Elbert and Hubbell, 2000). By incubating β-NGF microrods in 1 M NaCl, a statistically different 4-fold increase of β-NGF release was detected relative to the PBS groups, suggesting β-NGF entrapment within the PEGDMA microrods. Methods of protein immobilization and entrapment within hydrogel matrices via mesh size alterations has been used previously and can be useful for stabilization of delicate proteins (Li and Mooney, 2016). The encapsulation of β-NGF within the PEGDMA microrod was confirmed through theoretical calculations of mesh size (ξ) of 90% microrods, which was 1.865 nm compared to the hydrodynamic radius of β-NGF which has been reported to be between 2–4 nm (Stroh et al., 2003; Conovaloff et al., 2011). Furthermore, we also examined β-NGF-loaded microrods after 168 h of release and challenged them with 1 M NaCl. Interestingly, the 70% v/v microrod released more than 35% of the initially loaded protein compared to 90% v/v microrods. Given that the 70% PEGDMA microrods have a less dense matrix, the salts may have had more access to the protein within matrix inducing greater elution relative to the 90% microrods, which supports our theoretical calculations.

Although we were able to recover additional protein after loading and *in vitro* release through high salinity conditions, we aimed to see if additional physiochemical interactions could be occurring. PEGDMA has a number of ester bonds within its polymer chain and thus can form potential hydrogen bonds with drugs and cellular products (McNamee et al., 2007; Raju et al., 2018; Joshi et al., 2021). Therefore, to assess ester bond interactions we utilized ATR-FTIR to look at functional group spectral shifts before and after loading β-NGF. Specifically examining the ester peak at 1720 cm^−1^, a subtle shift from 1720 cm^−1^ to 1723 cm^−1^ was observed. A hypsochromic shift as such indicates either direct or indirect interactions with the etheric groups of the PEGDMA polymer chain. First, weakening of the polymer intermolecular reaction could be occurring as the polymer interacts with β-NGF. In this case, β-NGF could be forming additional hydrogen bonds with the etheric groups and thus weakening the intramolecular bond strength of PEGDMA. Given that NGF is known to form such bonds with its high affinity receptor TrkA and the lack of 1720 cm^−1^ in its secondary protein structure, β-NGF may be directly forming bonds with the PEGDMA main chain (Wiesmann et al., 1999; Paoletti et al., 2011; Covaceuszach et al., 2015). Second, recent works are starting to understand the strong interactions that occur between PEG and proteins (McNamee et al., 2007; Wu et al., 2014; Swartzlander et al., 2015). A similar contribution could be at play wherein the PEGDMA polymer chains and β-NGF are interacting to induce a conformational change of the protein. The current data suggest that β-NGF is having either a direct or indirect interaction within the PEGDMA mesh network that may be weakening the polymers intermolecular bond strength through hydrogen bond formation or potential PEG/protein interactions leading to physiochemical interactions potentially through hydrophobic domains of the protein. Despite this finding, we cannot definitively determine which factor is driving the high retention of β-NGF within the PEGDMA microrods, but it is most likely through a combination of factors. Nonetheless, our findings for high retention of β-NGF within the PEGDMA microrods is highly advantageous as it provides a delivery method to localize and stabilize protein within the fracture callus during endochondral fracture repair.

We next wanted to demonstrate that the loaded β-NGF retained its bioactivity with an *in vitro* assay using the TrkA expressing TF-1 cell line (Ma and Zou, 2013; Malerba et al., 2015). Importantly, this assay, as well as the release, was performed with 16,000 PEGDMA microrods, versus 100,000 used for the loading assay. This is because only 16,000 could effectively be aspirated in a 20 µL syringe used for the *in vivo* experiments. We calculated that approximately 61% of total protein loaded in 100,000 high concentration microrods was about 12.2 × 10^3^ ng or 0.12 ± 0.018 ng per microrod. Therefore, we used the highest calculation of 2000 ng that was loaded in 16,000 microrods and set that as our soluble β-NGF amount for all experiments in parallel. Here we found more than a 2-fold increase in cell number when TF-1 cells were treated with β-NGF conditioned media from microrods compared to media only. Neither the microrods themselves or soluble β-NGF led to a significant increase in cell number, although soluble β-NGF was trending higher than control. While we expected to see an increase in the soluble NGF control as well as the NGF-microrods, the response to β-NGF is not linear (Chevalier et al., 1994; Rende et al., 2000; Kemp et al., 2011) and perhaps the microrods protected the β-NGF from degradation as short-half life is a well-known limitation of β-NGF delivery (Alastra et al., 2021). Additionally, sucrose was added to the lyophilization solution containing the microrods to minimize aggregation. During loading, sucrose remains in the solution and presumably remains in the microrods along with β-NGF. A previous report has shown that disaccharides, including sucrose, can increase cell number and viability during short incubation times (i.e., <5 days) (Leong et al., 2017). Addtionally, potential interactions between β-NGF and PEGDMA may be playing a role in enhancing the bioactivity of the protein. Previous work looking at fibrin matrices containing heparin suspected a role of the heparin regulating the physiological function of β-NGF despite low affinity interactions (Sakiyama-Elbert and Hubbell, 2000). As shown from our data with the high retention of β-NGF within the microrod and physiochemical interactions occurring between the two, we speculate that the β-NGF that is released (>20 ng) has preserved bioactivity through interactions with the PEGDMA mesh network and/or the potential conformational changes induced via the microrods that enhance the proteins’ function relative to soluble β-NGF. Moreover, an important finding from this data is that the microrods loaded with β-NGF and non-loaded did not have any negative effects on cell viability. We have previously reported on PEGDMA microrod degradation and have shown that in similar conditions PEGDMA does not degrade in a 2-month span (Doroudian et al., 2014). Thus, we can conclude that the PEGDMA microrods are biocompatible as assessed by cell viability and can retain β-NGF bioactivity post-loading.

Next, we examined PEGDMA microrod localization during endochondral fracture repair in a murine fracture model and were able to histologically localize the PEGDMA microrods adjacent to the fracture callus at both 5- and 7-days post-injection. PEGDMA microrods were no longer visible after 14 days. While PEGDMA is not biodegradable in saline at this timescale, we speculate that mechanical breakdown or physical displacement of the rods may have accelerated their degradation since the fracture callus is undergoing dynamic remodeling and the animals were allowed to freely ambulate following microrod injection. (Rot et al., 2014). Alternatively, since acrylate-derivatized PEG hydrogels are chemically degraded through hydrolysis of the end group-ester (Burke et al., 2019; Stillman et al., 2020), the chemical microenvironment of the injury may have expedited degradation which has been observed *in vivo* for other ester-terminated polymers in similar microenvironments (Plecko et al., 2020; Yao et al., 2021). A limitation of the current study is the lack of longitudinal tracking of the microrods following injection. In our prior work, we incorporated iron oxide nanoparticles to create PEGDMA microrods that could be visualized by magnetic resonance imaging (MRI) in *ex vivo* cardiac tissue (Pinney et al., 2014); a similar technology could be explored for *in vivo* tracking in fracture repair (Kharbikar et al., 2021). Regardless, efficacy studies confirm that the PEGDMA microrods were localized in the fracture callus long enough to deliver the therapeutic payload and therefore are suitable for application in fracture callus injections of β-NGF.

Therapeutic efficacy of β-NGF delivery via PEGDMA microrods was tested in a murine tibial fracture model and quantitatively assessed using μCT analysis and quantitative histomorphometry. Our study validated that β-NGF loaded microrods enhanced endochondral fracture repair as evidenced by the reduction in cartilage volume and statistically significant increases in bone volume fraction (BVF), trabecular bifurcations (TB), and bone mineral density (BMD) compared to a single β-NGF injection. Notably, the woven-like bone morphology and minimal cartilage within the fracture callus further indicates a quicker cartilage to bone transition after injection. Importantly, the microrods produced values much closer to our prior work where NGF was administered by 3 injections over 3 days during the cartilagenous phase of healing (i.e., Day 7–9) which increased BVF to 27% ± 6.8, TB to 1,001/mm^3^ ± 235, and BMD to 291 mgHA/cm^2^ ± 38 (Rivera et al., 2020). Interestingly, the current studies show average values for BVF at 21% ± 4.2, TB to 942/mm^3^± 217, and BMD to 254 mgHA/cm^2^ ± 24. These values are statistically higher than the saline control and within the range of the prior study utilizing multiple injections.

We ascertain that the therapeutic benefit of PEGDMA microrods is through the stabilization of β-NGF and its localized release within the fracture callus. Presumably the dynamic microenvironment of the fracture callus and ambulation of the animals *in vivo* enhances protein delivery relative to the *in vitro* setting. However while intact, the microrods play an important role in protecting rapid clearance and degradation of soluble β-NGF within the fracture callus. Furthermore, the microrod may increase the therapeutic safety profile by minimizing the risk of off-target effects and toxicity compared to a single large bolus dose (Langer, 1998; US Food and Drug Administration Public Health Notification, 2008; Li and Mooney, 2016). In addition, such drug delivery strategies would be most optimal for situations involving delayed healing such as aging and diabetes that require longer dosing regimens. Although this was outside the scope of the study, it is an essential point for validation in future studies. Overall, high retention of β-NGF within the target tissue is a promising therapeutic approach (Hoare and Kohane, 2008; Wang et al., 2018). Further physiochemical optimization of the microrods for prolonged release may alter the release dynamics to further enhance the fracture repair healing response.

## Conclusion

We have demonstrated the feasibility of using PEGDMA microrods for β-NGF delivery and its therapeutic efficacy in a preclinical murine fracture model. We build on our previous data that showed repeated injections of β-NGF during fracture repair promotes endochondral fracture repair. Although that study used multiple injections, this study suggests a single local injection of PEGDMA microrods that contain 2000 ng of β-NGF may in fact be more beneficial than larger doses potentially due to extending the drug’s residence time and/or enhancing its biological activity. Given that the release is relatively low (>20 ng) and most of the protein is retained within the microrod, this delivery method is clinically relevant and could decrease the known hyperalgesic effects. Thus, limiting the total amount of β-NGF released within the target tissue could potentially reduce the pain experienced by subjects. From a therapeutic standpoint, reducing the pain and number of injections is critical given that increased stress can lead to delayed fracture healing (Haffner-Luntzer et al., 2019). To deliver β-NGF, we utilized PEGDMA hydrogels, as PEG and PEG-conjugates have been FDA approved in the past for several products from tissue adhesives to particle based drug and gene delivery systems (Bré et al., 2013; Suk et al., 2016; Moncalvo et al., 2020). To that end, the use of injectable β-NGF loaded PEGDMA microrods validates a novel and translational therapeutic approach for improving bone fracture repair.

## Data Availability

The raw data supporting the conclusion of this article will be made available by the authors, without undue reservation.
