# Supplementary figures and images for: Encapsulation of β-NGF in injectable microrods for localized delivery accelerates endochondral fracture repair
Source: Front Bioeng Biotechnol. 2023 May 22;11:1190371. doi: 10.3389/fbioe.2023.1190371 (PMC10241161; doi:10.3389/fbioe.2023.1190371)

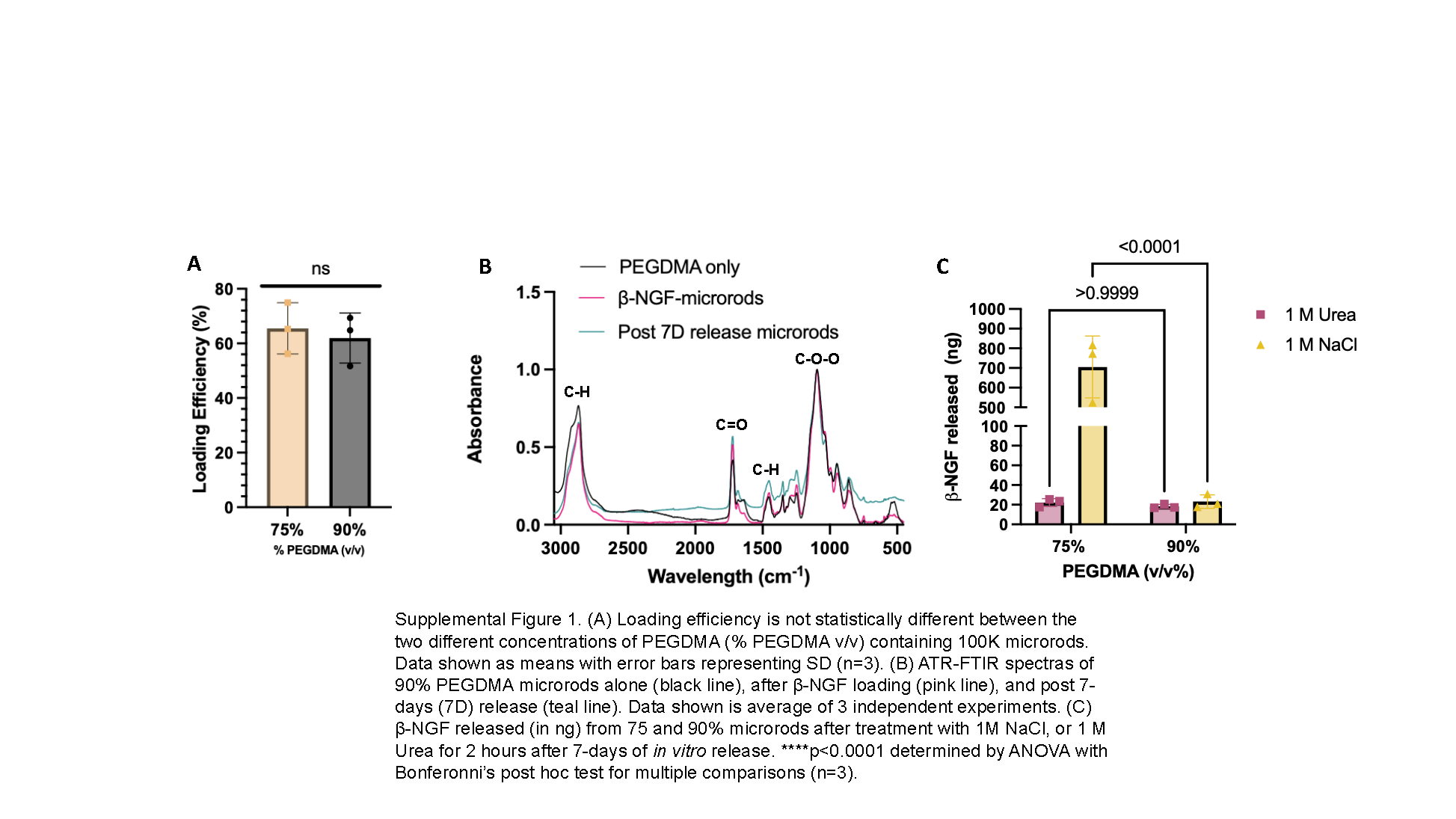

Supplement: Supplementary file 1 [file Image1.tiff]

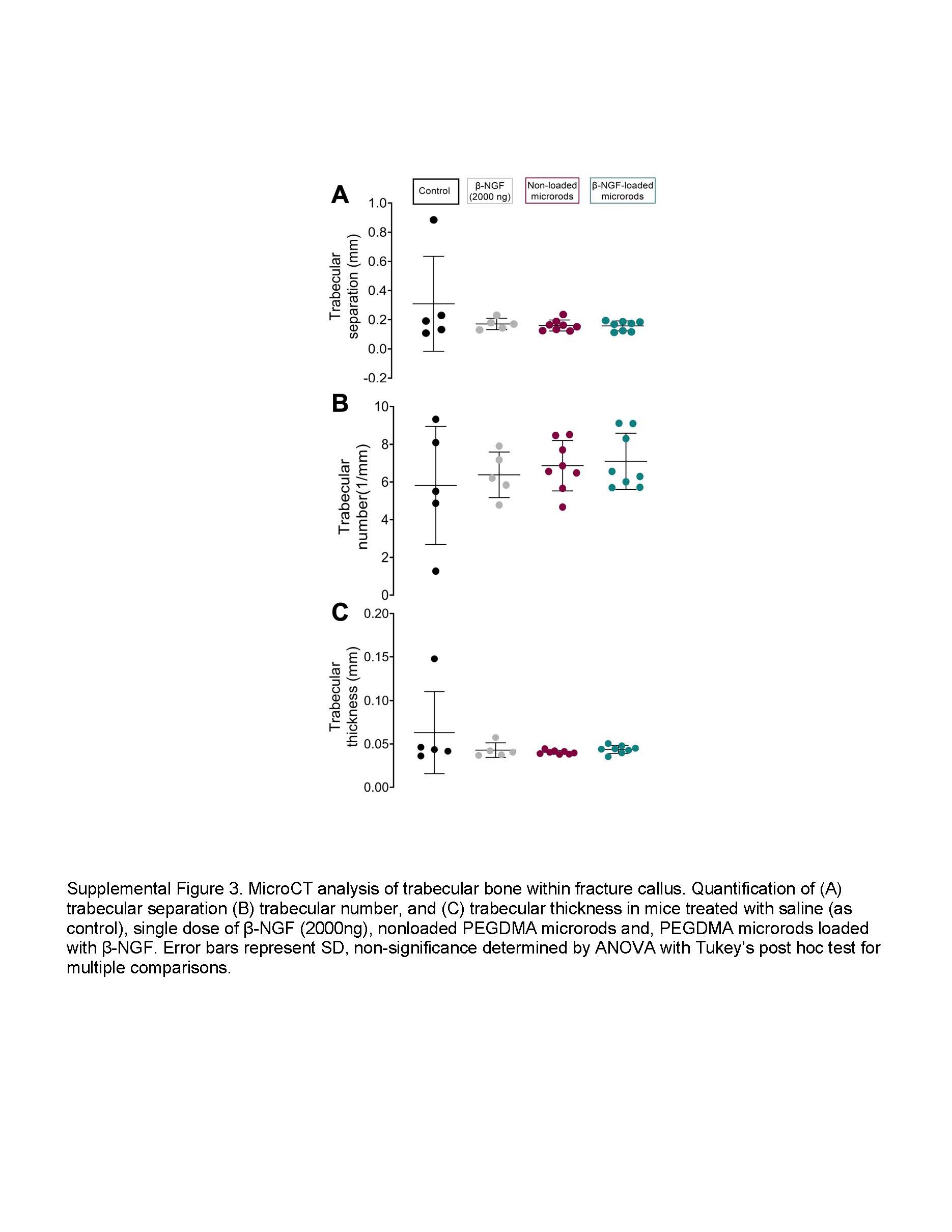

Supplement: Supplementary file 2 [file Image3.jpg]

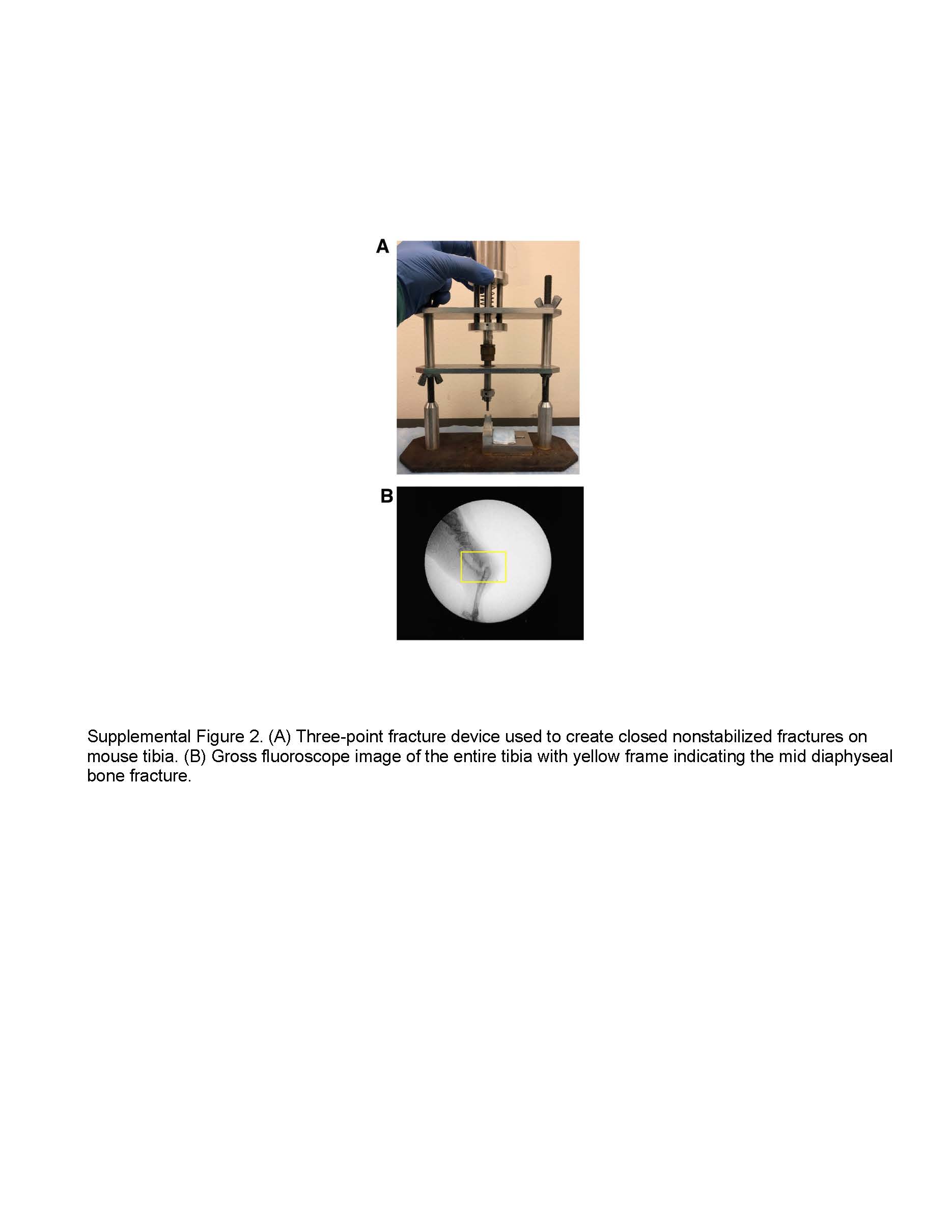

Supplement: Supplementary file 3 [file Image2.jpg]
